# The effect of oral ferrous sulfate use in different posology on laboratory values and clinical findings related to iron metabolism in anemia due to iron deficiency

**DOI:** 10.1097/MD.0000000000044084

**Published:** 2025-08-29

**Authors:** Merve Yüksel, Merve Aydoğan, Özlem Doğan, Meltem Kurt Yüksel

**Affiliations:** a Department of Hematology, Ankara University School of Medicine, Ankara, Turkey.

**Keywords:** ferrrous sulfate, hemoglobin, hepcidin, iron deficiency anemia

## Abstract

Iron deficiency anemia (IDA) is a common general health problem in daily clinical practice. A large amount of iron in the body is used for hemoglobin (Hb) synthesis and iron is critical for many biological functions such as cell proliferation, energy production, DNA synthesis and respiration. In recent years, with a better understanding of iron metabolism; it has led to the need to review treatment regimens in the treatment of IDA. Based on these data, we aimed to evaluate the efficacy of oral ferrous sulfate treatment with different doses and posology, its relationship with hepcidin, treatment compliance and gastrointestinal side effects in premenopausal women diagnosed with IDA. Our study was a prospective observational study and included premenopausal female patients aged 18 to 50 years diagnosed with IDA. The patients, who were started on oral ferrous sulfate treatment with different posology and dose, were divided into 3 groups as 2*1 day in the first group, 1*1 day in the second group and 1*1 every other day in the third group and their treatment was completed for 3 months. Changes in Hb and hepcidin were evaluated before treatment and in the second week, and changes in ferritin, transferrin saturation %, total iron binding capacity, and Hb were evaluated in the 3rd month. Significant Hb increase was observed at the end of the second week (*P* < .01) and the mean Hb increase was 1.38 ± 1.04 and 1.03 ± 0.48 and ≥1 g/dL in the first and second groups, respectively, while it was 0.69 ± 0.36 and < 1 g/dL in the group given every other day (*P* = .020, *P* = .019, respectively). At the end of the 3rd month, there was a significant increase in Hb level in all 3 groups (*P* < .001) and Hb increase was similar between the groups (*P* > .05). When the change in ferritin was analyzed, it was observed that ferritin was statistically significantly higher in the first group (2*1) compared to the second and third groups (*P* < .05). There was no difference between group 2 and group 3 in terms of ferritin change values (*P* > .05). The increase in transferrin saturation % and decrease in total iron binding capacity were similar in all 3 groups at the end of treatment (*P* > .05). The change between the second week and baseline hepcidin was observed most in the second group (*P* = .024) and the change in hepcidin was similar between the 3 groups (*P* = .708). Gastrointestinal side effects were observed more in the first group receiving 2*1 than in the second and third groups (*P* < .05). Patients in groups 1 and 2 showed a similar increase in appetite and weight at the end of treatment (*P* > .05), whereas no increase in appetite and weight was observed in group 3. In our study, Hb increase of ≥ 1 g/dL in the first and second treatment groups and < 1 g/dL in the third treatment group was observed at the end of the second week; at the end of treatment, anemia improved significantly and Hb increase was similar in all 3 groups. However, due to the gastrointestinal side effects that were significant in the first group, we think that 1*1 daily or 1*1 every other day instead of 2*1 would be more appropriate. In addition, we think that serial hepcidin measurements would give a better idea about the kinetics. Larger studies may emphasize the importance of alternative iron treatment regimens.

## 1. Introduction

Iron deficiency (ID) and iron deficiency anemia (IDA) is a common public health problem in daily clinical practice.^[1]^ It affects more than 1.2 million people worldwide and is the leading cause of anemia among all anemias. Especially in developing countries, iron (Fe) deficiency significantly affects the quality of life of children and premenopausal women.^[2]^

ID is defined as a decrease in iron stores in the body, especially in hepatocytes and macrophages, with or without anemia. According to the World Health Organization (WHO), the hemoglobin (Hb) limit for anemia is < 13 g/dL in men, <12 g/dL in women and < 11 g/dL in pregnant women.^[3]^ The etiology of ID in developing countries is usually due to inadequate dietary Fe intake, malnutrition and chronic blood loss caused by intestinal parasites.^[4]^ In developed countries, the best known causes are vegetarian diet, malabsorption and chronic blood loss due to heavy menstrual bleeding. In men and the elderly, chronic blood loss due to benign lesions in the gastrointestinal system (GIS) and cancer can also lead to Fe deficiency.^[5,6]^

Systemic iron balance in the body is under the control of the hepcidin mechanism. Hepcidin is a peptide hormone produced in the liver. It functions as an acute phase reactant that regulates Fe absorption from enterocytes and Fe release from splenic macrophages into plasma.^[7,8]^ Hepcidin levels increase in the presence of increased plasma Fe levels, inflammation or infection. Increased erythropoiesis, Fe deficiency and tissue hypoxia lead to decreased levels of hepcidin. Intestinal epithelial cells and macrophages of the reticuloendothelial system use the ferroportin (FPN) system to transport iron into plasma. Increased hepcidin in serum binds to the FNP receptor and blocks the absorption of dietary iron. On the contrary, inhibition of hepcidin increases the release of iron from hepatocytes and macrophages into plasma and its absorption from enterocytes.^[9]^ In addition, there is a negative correlation between hepcidin and erythroferron (ERFE) in iron hemostasis. ERFE is a glycoprotein hormone produced by erythroblasts in response to erythropoietin, a direct liver acting erythropoietin that suppresses hepcidin to promote mobilization of stored iron and absorption of dietary iron. So far, there are few studies in humans on the role of ERFE in IDA, and most studies have been conducted in animals. Defining the mechanisms of this dysregulation is vital for understanding the pathogenesis of conditions associated with impaired iron metabolism and erythropoiesis activity.^[10,11]^

Oral and intravenous routes are among the treatment options in the treatment of IDA. Oral iron therapy in adults includes ferrous sulfate, ferrous gluconate and ferrous fumarate. Among these, ferrous sulfate is the most commonly used oral treatment agent.^[12]^ Therapeutic doses may vary between 100 and 200 mg depending on the severity of symptoms, ferritin level and GIS side effects. According to recent studies, oral Fe treatment acutely increases serum hepcidin levels, which decreases the absorption of subsequent doses of Fe. Due to the fact that Fe homeostasis is under the control of the hepcidin protein and the negative effects of high hepcidin levels; it has led to the need to determine the most appropriate dose and posology in the treatment of IDA and to reconsider the treatment regimens. Based on this, previous studies in non-anemic, Fe deficient young women have shown that oral ferrous sulfate therapy given every other day instead of every day, in single doses instead of divided doses, and in low doses improves the efficacy and tolerability of treatment.^[13,14]^

Based on these data, in this study, we aimed to evaluate the efficacy of oral ferrous sulfate treatment at different doses and posology, its relationship with hepcidin, treatment compliance and gastrointestinal side effects in premenopausal women diagnosed with IDA.

## 2. Materials and methods

### 2.1. Protocol of the study

Our study was a prospective observational study and included premenopausal female patients aged 18 to 50 years with a diagnosis of IDA who applied to Ankara University Faculty of Medicine Hematology and Internal Medicine outpatient clinics between October 2019 and February 2020.

### 2.2. Inclusion and exclusion criteria

#### 2.2.1. Study inclusion criteria

- 18 to 50 years old premenopausal female patients.
- Diagnosed with IDA.
- Ferritin < 30 mg/L, Hb < 12 g/dL and C reactive protein < 5 mg/L.

#### 2.2.2. Study exclusion criteria

- Anemia other than ID and treatment with any iron product other than ferrous sulfate within the last 3 months.
- Active infection, renal and heart failure, rheumatologic disease, inflammatory bowel disease, history of malignancy.
- Celiac disease, autoimmune atrophic gastritis.
- Proton pump inhibitors, H2 receptor blockers, levothyroxine, levodepo, quinolone group antibiotics, tetracyclines.
- Pregnant and lactating women.
- Blood donors in the last 4 months.
- Abnormal menstrual cycle.

### 2.3. Data collection

Ankara University Faculty of Medicine Hematology and Internal Medicine outpatient clinics and 90 women diagnosed with IDA who met the study criteria were included in the study. The study was explained to all participants and the participants who signed the voluntary consent form were included in the study. The patients who were included in the study and started oral ferrous sulfate treatment with different posology and dose by their physicians were divided into 3 groups.

Treatment group I (n = 28): 2*1 160 mg/daily ferrous sulfate

Treatment group II (n = 30): 1*1 80 mg/daily ferrous sulfate

Treatment group III (n = 32): 1*1 80 mg/alternate day ferrous sulfate

Patients were followed up for 3 months, called every 2 to 3 weeks to check the regular use of their medications and were called for followup at the second week and at the end of the 3rd month to evaluate treatment responses. A complete blood count was ordered at the 2nd week to evaluate Hb elevation and ferritin, transferrin saturation (TSAT%) and complete blood count were ordered at the 3rd month to evaluate iron stores.

In addition, routine blood tests were requested from the patients before treatment and at the 2nd week controls. For the measurement of hepcidin level, extra blood was taken from peripheral venous blood into 1 biochemistry tube, venous blood samples were centrifuged at 1000 ×g for 20 minutes and serum was separated and the sera obtained were placed in Eppendorfs and stored at −20°C until measurement.

During the study period, the COVID-19 pandemic, which started in China in December 2019, started to be seen in our country as of March 2020 and continued for more than one year. Due to the COVID-19 Pandemic and restrictions, although 32 of the 90 patients completed their treatment for 3 months, the 3rd month data could not be reached, so the 2nd week and 3rd month evaluations were made with 58 patients’ data. At the end of the treatment, patients filled out a side effect form to evaluate the side effects that developed and those who could not come to the hospital due to the pandemic were evaluated by phone call.

### 2.4. Ethics committee approval and budget

Our study, which was approved by the Ankara University Faculty of Medicine Clinical Research Ethics Committee (Approval date: September 18, 2019, Decision no: 13-999-19), was supported by Ankara University Scientific Research Projects fund (Project Number 20L0230012). On the same date (September 18, 2019), approval for the observational drug study was obtained from the Ministry of Health, Turkish Medicines and Medical Devices Agency.

### 2.5. Statistical analysis

Mean standard deviation, median minimum and maximum values were given in descriptive statistics for continuous data, and number and percentage values were given in discrete data. Kolmogorov-Smirnov test was used to examine the suitability of the data for normal distribution.

T test was used to compare the difference between 2 groups of independent variables in continuous data showing normal distribution, and one-way analysis of variance was used to compare independent variables with more than 2 groups; Mann–Whitney *U* test was used to compare the difference between 2 groups of independent variables in data not conforming to normal distribution, and Kruskal Wallis Analysis of Variance was used to compare independent variables with more than 2 groups.

Repeated measures analysis of variance was used to examine the differences in basal, 2nd week and 3rd month follow ups of normally distributed data. Friedman test was used to examine the differences in basal, 2nd week and 3rd month follow-ups of non-normally distributed data. Friedman’s multiple comparison test was used to analyze the timing of the difference. Wilcoxon test was used to examine the differences in baseline and 3-month follow ups of data that did not show normal distribution.

Chi-square and Fisher’s exact test were used in group comparisons of nominal variables (cross-tabulations). IBM SPSS Statistics 20 program was used in the evaluations and *P* < .05 was accepted as the statistical significance limit.

## 3. Findings

A total of 90 premenopausal women aged 18 to 50 years, 28 in the first treatment group, 30 in the second treatment group and 32 in the third treatment group, were included in our study. The mean age of the patients in the first treatment group was 35.61 ± 10.49, 36.83 ± 8.33 in the second treatment group and 36.78 ± 8.24 in the third treatment group.

The baseline laboratory values of the patients in the 3 treatment groups and their comparison are presented in Table 1. Accordingly, no difference was found between the baseline laboratory values of the patients in the first, second and third treatment groups (*P* > .05).

**Table 1:** Comparison of baseline laboratory values in treatment groups.

Baseline	Group 1 (n = 28)	Group 2 (n = 30)	Group 3 (n = 32)	Test statistics	*P* *
	Med ± SD Median (min–max)	Med ± SD Median (min–max)	Med ± SD Median (min–max)		
CRP (mg/L)	1.75 ± 1.43 1.15 (0.2–4.5)	2.24 ± 1.58 1.9 (0.2–4.8)	2.01 ± 1.46 1.85 (0.3–4.5)	χ^2^ = 1.809	.405
Vitamin B12 (pg/mL)	320.46 ± 45.81 307.5 (250–475)	302.37 ± 41.16 292.5 (232–418)	321.66 ± 61.56 310 (258–529)	χ^2^ = 3.842	.146
Folate (ng/mL)	9.36 ± 2.69 8.3 (5.8–16.2)	8.65 ± 3.24 7.7 (5.3–19.7)	9.41 ± 4.10 8.6 (5.5–24)	χ^2^ = 2.731	.255
TSH (µIU/mL)	2.11 ± 1.09 1.87 (0.47–4.52)	2.02 ± 0.80 2 (0.66–4.08)	1.76 ± 0.63 1.65 (0.78–3.70)	χ^2^ = 1.714	.424
Hemoglobin (g/dL)	9.72 ± 1.33 9.6 (7.1–11.8)	9.98 ± 1.38 10.4 (5.7–11.7)	9.98 ± 1.47 9.8 (7–11.8)	*F* = 0.252	.778
Plasma Fe (µg/dL)	25.82 ± 18.24 20 (10–96)	20.63 ± 8.33 21 (8–38)	26.69 ± 16.30 18 (9–67)	χ^2^ = 1.069	.586
TSAT (%)	5.54 ± 3.46 4 (2–16)	4.90 ± 1.83 5 (2–9)	6.09 ± 3.59 5 (2–17)	χ^2^ = 0.830	.660
Ferritin (ng/mL)	4.74 ± 4.43 3.2 (1.6–18.4)	3.00 ± 1.05 2.8 (1.4–5.5)	3.48 ± 1.87 2.9 (1.4–8.8)	χ^2^ = 1.558	.459
TIBC (µg/dL)	457.29 ± 52.07 459 (335–567)	447.17 ± 59.47 443.5 (351–626)	442.19 ± 49.68 449 (349–533)	*F* = 0.602	.550
PLT (10^9^/L)	341.11 ± 108.23 317.5 (153–649)	384.93 ± 128.32 362 (216–880)	317.25 ± 76.87 304 (182–473)	χ^2^ = 5.063	.080
Hepcidin (pg/mL)	121.92 ± 79.41 104.1 (54.9–453.7)	131.35 ± 65.47 114 (64.2–416.6)	115.95 ± 100.59 93.1 (60.7–636.9)	χ^2^ = 6.515	.038

ANOVA = analysis of variance, CRP = C reactive protein, PLT = platelet, TIBC = total iron binding capacity, TSH = thyroid stimulating hormone.

* Kruskal–Wallis variance analysis/(ANOVA).

When subanalysis of basal hepcidin was performed.

There was no correlation between baseline Hb, Ferritin and TSAT% and baseline Hepcidin values (*P* > .05) (Table 2).

**Table 2:** Correlation between baseline hepcidin values and baseline Hb, baseline ferritin and TSAT% values.

	Baseline hepcidin	
	*r* *	*P*
Baseline Hb	−0.010	.931
Baseline ferritin	−0.028	.801
Baseline TSAT %	−0.053	.629

Hb = hemoglobin, TSAT = transferrin saturation.

* Spearman correlation coefficient.

The baseline and posttreatment 2nd week and 3rd month blood parameter levels of female patients with IDA in 3 treatment groups were compared and changes were analyzed. Since some patients who used iron treatments regularly for 3 months but could not come to the hospital for the 3rd month control due to the COVID-19 Pandemic, only patients with 3rd month data could be evaluated. Accordingly, the data of 13 female patients in the first treatment group, 23 in the second treatment group and 22 in the third treatment group were evaluated.

**Figure 1.**
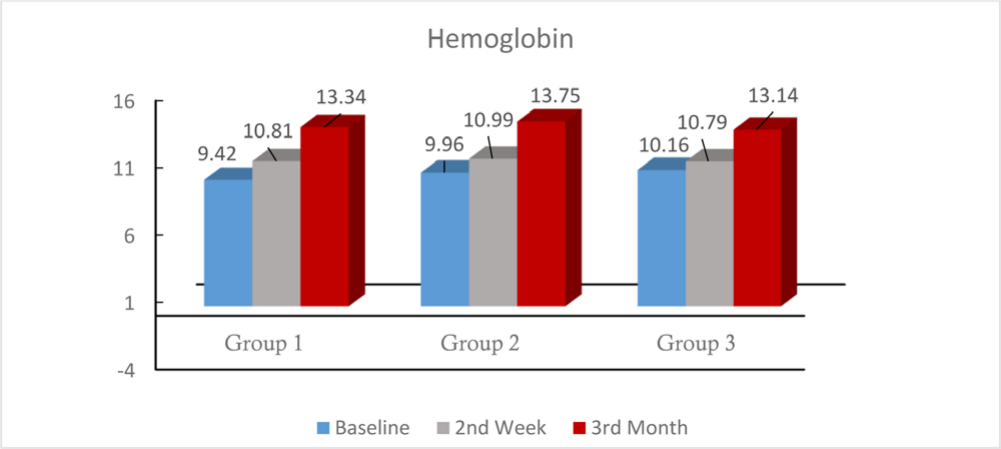
Hb change according to treatment groups.

Baseline Hb levels and Hb increase in the treatment groups are shown in Figure 1. There was statistical significance in Hb levels before and after treatment in all 3 groups (*P* < .001). At the end of the treatment, anemia of the patients in all 3 groups improved, while anemia of 2 patients, one each in the group taking twice a day and alternate day, did not improve. There was no significant difference between the 3 groups in the increase in Hb values at baseline, 2nd week and 3rd month (*P* > .05).

When Hb change according to time was analyzed between the groups, there was a difference in Hb changes from baseline to week 2 (*P* < .05) and Hb change from baseline to week 2 was significantly lower in group 3 patients compared to both group 2 and group 1 patients (*P* = .020, *P* = .019, respectively), while there was no difference between group 1 and group 2 (*P* > .05). Hb changes from baseline to month 3 were not different in the 3 treatment groups (*P* > .05). As a result, among the 3 treatment groups, the Hb increase in the 2nd week compared to baseline was the lowest in the group receiving alternate day, while the change in the 3rd month was similar in all 3 groups (Table 3).

**Table 3:** Hb changes from baseline to 2nd week and from baseline to 3rd month in treatment group.

Hemoglobin changes	Group 1 (n = 13)	Group 2 (n = 23)	Group 3 (n = 22)	Test statistics	P*
	Med ± SD Median (min–max)	Med ± SD Median (min–max)	Med ± SD Median (min–max)		
Week 2 Hb difference compared to baseline	1.38 ± 1.04 1 (−0.10 to 3)	1.03 ± 0.48 0.9 (0.4–2.6)	0.69 ± 0.36 0.60 (0.2–1.7)	χ^2^ = 7.537	.023
3rd month Hb difference compared to baseline	3.92 ± 1.95 3.4 (0.1–7.3)	3.79 ± 1.46 3.5 (1.8–7.8)	2.98 ± 1.36 2.9 (0.5–5.7)	χ^2^ = 4.041	.133

Hb = hemoglobin.

**Figure 2.**
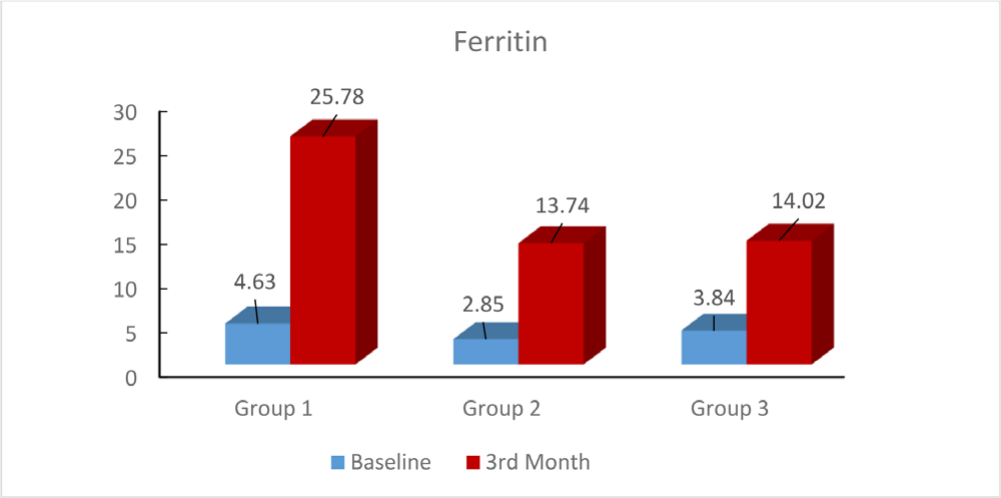
Ferritin change according to treatment groups.

At the end of 3 months of treatment, there was a significant increase in TSAT% (*P* < .001) and ferritin (*P* < .01) in all 3 groups. Accordingly, ferritin values of patients in group 1 were significantly higher than those of women in both group 2 and group 3 at the end of 3 months (*P* = .001, *P* = .005, respectively) (Fig. 2).

When the change in ferritin was analyzed; the highest increase was observed in the first treatment group receiving twice daily (*P* = .003, *P* = .003, respectively).

**Figure 3.**
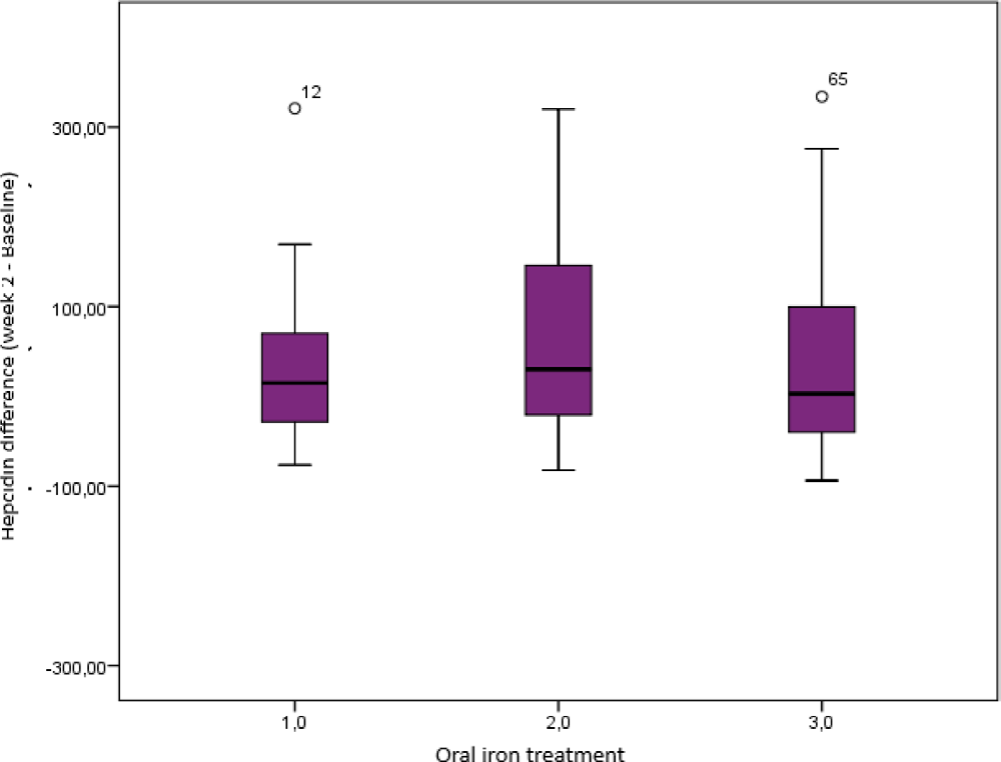
Changes in hepcidin over time.

There was no difference between the baseline and 2nd week Hepcidin values of the patients in the first and third treatment groups (*P* > .05), whereas there was a difference in the second treatment group (*P* < .05). The change in hepcidin from baseline to week 2 was observed most in the 2nd group (Fig. 3). The mean hepcidin changes from baseline to week 2 were 22.14 ± 137.70 in the first treatment group, 62.37 ± 109.38 in the second treatment group and 42.27 ± 118.03 in the third treatment group and were similar in the 3 treatment groups (*P* = .708).

At week 2, hepcidin changes between baseline and week 2 were examined in patients with Hb increase < 1 g and  ≥ 1 g. There was a difference between the changes in patients with Hb increase < 1 g (*P* = .025) and a significant increase in hepcidin. There was no difference between baseline and 2nd week hepcidin values in patients with Hb increase ≥ 1 g (*P* > .05).

**Figure 4.**
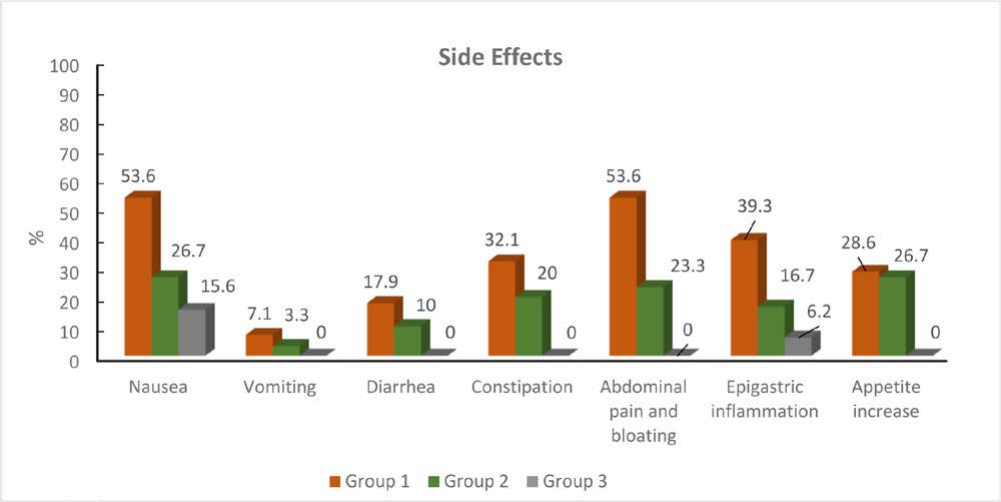
Side effect rates according to treatment groups.

Figure 4 shows the side effects seen in patients at the end of 3 months of treatment and their rates. Accordingly, the highest number of gastrointestinal side effects was observed in the first group receiving twice daily oral Fe and no patient discontinued treatment due to side effects. In all groups with side effects, only G1-2 side effects were observed, no G3-4 side effects were observed and there was no difference between the groups in terms of side effect grades (*P* > .05).

While no weight gain was observed at the end of the treatment in the third group patients, weight gain was observed at the end of the treatment in patients in groups 1 and 2 and there was no significant difference in the amount of weight gain (*P* > .05) (Table 4).

**Table 4:** Comparison of the amount of weight gain in treatment groups.

	Group 1 (n = 28)	Group 2 (n = 30)	Group 3 (n = 32)	Test statistics	*P* *
	Med ± SD Median (min–max)	Med ± SD Median (min–max)	Med ± SD Median (min–max)		
Weight increase Amount	1.29 ± 2.11 0 (0–6)	0.90 ± 1.69 0 (0–5)	– 0 (0–0)	χ^2^ = 0.475	.491

* Kruskal–wallis variance analysis.

When Hb increase was analyzed in patients with weight gain compared to those without weight gain, it was observed that Hb increase was higher in patients with weight gain (*P* < .01) (Table 5).

**Table 5:** Hb changes compared to baseline in patients with and without weight gain.

Hb changes	No weight gain	Weight gain	Test statistics	P*
	Med ± SD Median (min–max)	Med ± SD Median (min–max)		
3rd month Hb difference compared to baseline	3.22 ± 1.54 3.1 (0.1–7.8)	4.52 ± 1.30 5.1 (1.8–5.9)	U = 139.5	.004

Hb = hemoglobin.

## 4. Discussion

In the treatment of IDA, oral Fe preparations are generally used as the first choice because they are effective, inexpensive and safe. However, there is variation among clinicians in the appropriate dose of drugs for the treatment of ID. The British Society of Gastroenterology recommends the use of ferrous sulfate as first-line therapy for iron replacement because it is affordable, has high bioavailability, is widely available in a variety of forms and has been shown to effectively replace iron stores.^[15]^ For patients with IDA, treatment should focus on improving iron stores and normalizing Hb. Although treatment guidelines generally recommend 100 to 200 mg of elemental Fe in single or divided doses daily, in recent years, with a better understanding of iron metabolism, lower doses have been shown to be more effective and better tolerated.^[16]^ This is because the absorption of nonheme iron is low and decreases by 5% to 28% when fasting and by 2% to 13% when ingested with food. High doses may cause free oxygen radical-mediated toxicity of unabsorbed iron to the intestinal mucosa, worsening gastrointestinal symptoms and decreasing adherence to treatment.^[17]^ In addition, hepcidin is the most important molecule regulating iron metabolism in mammals, and a slight increase in serum iron activates hepcidin to limit iron absorption.^[9,18]^ A recent study examined daily, once-daily split and alternate day oral iron dosing regimens in women with moderate anemia. The results were superior to iron therapy alternate day and reported increased fractional iron absorption.^[19]^

Therefore, the primary aim of this study was to compare the efficacy of oral iron therapy at different doses and posology in IDA, and the secondary aim was to investigate the relationship with hepcidin, whether side effects were minimized and treatment compliance.

In this study, we examined the efficacy of oral ferrous sulfate with 3 different treatment modalities in women with IDA. The results showed that daily single dose and alternate day dose treatment significantly contributed to the increase in Hb levels. Our study confirms and extends our previous short-term studies. Although this study is not the first study to investigate the efficacy of iron therapy variations, it includes detailed clinical data such as side effects, iron therapy, and the relationship between Hb increase and weight gain, and it contains new results compared to other studies conducted with women with IDA.

When the literature is examined; in a study conducted by Kaundal et al patients with IDA were randomized to receive 60 mg 2*1 and 120 mg alternate day. At the end of 3 weeks, Hb increase was found to be 1.6 ± 1.2 and 1.1 ± 0.9; at the end of 6 weeks, 2.9 ± 1.7 and 2.0 ± 1.3, respectively, and Hb increase was faster in the group taking every day than in the group taking alternate day. However, they also emphasized that the mean Hb increase in the group receiving alternate day at the end of 6 weeks was not significantly different from the group receiving alternate day at the end of 3 weeks. The median increase in Hb levels was similar in mild anemia, whereas in moderate-to-severe anemia, patients receiving 120 mg elementary iron daily had a significantly greater mean Hb increase than patients receiving 120 mg elementary iron alternate day.^[20]^ In our study, the mean Hb increase at the end of week 2 was ≥ 1 g/dL in the group given twice and once daily, whereas the Hb increase was slower in the group given alternate day, and the mean Hb increase remained < 1 g/dL at week 2. In addition, we think that the Hb increase at week 3 in our study will be similar to this study. Finally, at the end of the 3rd month, we found that the anemia of the patients in all 3 groups improved and the mean Hb reached was similar, in addition, it was slightly higher in the group that received once every day (*P* = .052).

In another study conducted by Oflas et al in 2019 with 150 female patients with IDA, patients were divided into 3 groups and given 80 mg elementary Fe treatment every day, twice a day and once and alternate day, and at the end of 1 month of treatment, Hb increase was found at a similar rate in all 3 groups (≥2 g/dL). Ferritin increased the most in the group given twice a day and was statistically significant.^[21]^ Similar to these studies, in our study, we observed statistically significant increases in ferritin, TSAT % and serum Fe levels in all 3 groups at the end of the 3rd month, but the highest increase in ferritin was observed in the group given twice daily. We found a similar increase in ferritin in the group given once a day and alternate day.

Hepcidin is an important regulatory molecule in iron metabolism. In our study, hepcidin levels increased in 3 groups after 2 weeks of treatment and the highest increase was observed in the second group and no significant difference was observed between the 3 groups. In a study conducted by Moretti et al in patients with iron deficiency, they showed that 60 to 240 mg elementary ferrous sulfate treatment triggered an increase in hepcidin for up to 48 hours and limited the absorption of subsequent doses.^[12]^ ERFE is also an important protein in iron hemostasis. An important study in the literature reflecting the correlation between Hepcidin and ERFE and laboratory parameters in patients with IDA is the study by Talawy et al. In this study, plasma ERFE levels were higher than normal in all cases and there was a negative correlation with plasma hepcidin. ERFE was found to be significantly associated with indicators of iron metabolism (Hb, ferritin, mean corpuscular volume, mean corpuscular hemoglobin [MCH] and MCH concentration [MCHC]) in both ID and IDA patients. In both ID and anemia cases, there was a nonsignificant weak negative correlation with the above-mentioned parameters, while a positive correlation was found with platelet count.^[10]^ Determining the correct role and relationship between ERFE and hepcidin and different parameters will help in differentiating and classifying the stages of ID and various types of anemia.

Another study by Stoffel et al in which patients received 60 mg elementary fe every 14 days and alternate day for 28 days was limited by the absence of clinically relevant outcomes such as side effects. In this study, patients in the second group were given doses of 100 mg or 200 mg of iron with stable iron isotopes 57Fe, 58Fe and 54Fe over a period of time. Fractional iron absorption was higher in the second group (21.8% vs 16.3%) and hepcidin levels were higher in the alternate day group at the end of treatment.^[13]^ Kaundal et al evaluated the increase in Hb in patients with IDA treated with 120 mg elementary fe every day for 3 weeks and 120 mg fe alternate day for 6 weeks; they emphasized that plasma hepcidin change at the end of the 1st week was slightly higher in the group receiving alternate day (0.75 vs 1.2) but there was no statistical difference.^[20]^ In our study, on the other hand, more hepcidin change was found in the once-daily group and this change was not found to be significant. When the plasma hepcidin change of patients with Hb change < 1 g/dL and ≥ 1 g/dL at week 2 was examined, a significant increase was found in patients with < 1 g/dL compared to baseline. This hepcidin change may explain the lower and slower increase in Hb.

One of the important problems seen with oral iron preparations during treatment is gastrointestinal side effects. Oral ferrous preparations may cause nausea, vomiting, indigestion, metallic taste, constipation, diarrhea or dark stools.^[22]^ A possible explanation is that unabsorbed iron may cause intestinal inflammation and iron-induced microbiota changes.^[23]^ These common side effects may limit patient compliance with treatment. Previous studies have investigated associations between high-dose iron replacement and GI side effects.^[24,25]^

When we look at the literature, it has been reported that GI side effects are less common when oral iron is given in lower doses as a single dose rather than in divided doses. In the study conducted by Oflas et al the most GI side effects were observed in the group receiving twice daily; nausea and abdominal pain in 19.4%, diarrhea in 37.5%, and epigastric burning in 27.3%.^[21]^ In another study conducted in 2017, similarly, GI side effects were observed at a higher rate in the group receiving every day and 2 divided doses per day compared to the group receiving alternate day and single dose per day.^[13]^ Similar to the literature, in our study; the most GI side effects were observed in the group receiving twice daily. These findings led us to realize that increasing doses of iron may cause more side effects when given all at once in the hope of increasing the efficacy of the treatment. Oral iron supplements are recommended to be taken in low doses once daily after meals to avoid GI side effects and subsequent noncompliance. The amount can then be adjusted according to the clinician’s choice. Patients should try to take iron therapy on an empty stomach. To facilitate absorption, it should be taken with an average-sized glass of orange juice.

Although appetite and weight gain are observed in some patients in clinical practice during iron therapy, the information on this subject is not sufficient and clear. Bünger et al^[26]^ stated in their study that there should be enough iron for a normal appetite, IGF-1 (insulin growth factor-1), triiodothyronine secretion and glucose utilization, so when ID is eliminated, there will be an improvement in appetite.^[27]^ When we look at the studies on this subject in the literature; Yokuş et al in their study conducted in 2016; an average increase of 2.8 kg was observed after 3 months of oral fe treatment in women with IDA.^[28]^ In the study by Solmaz et al 1 month after intravenous Fe treatment was administered to 42 female patients with DEA, it was found that 52.4% patients had increased appetite and the mean weight increased from 69.6 to 70.7 kg.^[29]^ Similarly, in our study, at the end of 3 months, 28.6% and 26.7% of the first and second groups had increased appetite, 1.29 and 0.9 kg weight gain, respectively, whereas no increase in appetite or weight gain was found in the group receiving alternate day. Therefore, all these results, including our study, show that by replacing deficient iron, which is critical for cell proliferation, energy production, DNA synthesis and many respiratory biological functions in the body, physiological functions improve, patients’ complaints decrease and they also improve functionally.

The most important limitation of our study is that the COVID-19 pandemic started in December 2019 during the period of our study. Due to the pandemic continuing for more than one year and restrictions, 1 third of the patients in the study could not come to the 3rd month blood control despite completing their treatment. Therefore, the COVID-19 pandemic may have affected patient follow-up and data collection, leading to missing data for the 3rd-month assessment, which may have affected our study results. We plan to overcome this limitation with a new study that we will plan with a larger patient cohort with regular follow-ups. Another limitation is the relatively small sample size.

The main advantages of our study include longer duration of iron treatment, long-term follow-up data collection, inclusion of patients with anemia rather than just ID, widely accepted tolerable iron dose measurement and clinically relevant results. Furthermore, the assessment of inflammation using markers such as C reactive protein, the status of participants at the start of treatment, contributed to the exclusion of inflammatory processes and hence healthy results in ferritin values and changes. The main finding of our study is that instead of twice a day due to the significant GIS side effects in the first group, once a day or alternate day treatment should be recommended with similar treatment success, since Hb increase is slower in the alternate day group, we think that the 3rd or 4th week would be more appropriate to evaluate the Hb response, in addition, we think that serial hepcidin measurements would give a better idea about the kinetics. Larger studies may emphasize the importance of alternative iron treatment regimens.

## Author contributions

**Conceptualization:** Merve Yüksel, Özlem Doğan, Meltem Kurt Yüksel, Merve Aydoğan.

**Data curation:** Merve Yüksel, Merve Aydoğan, Meltem Kurt Yüksel.

**Formal analysis:** Merve Yüksel, Özlem Doğan.

**Funding acquisition:** Merve Yüksel, Meltem Kurt Yüksel.

**Investigation:** Merve Yüksel, Merve Aydoğan, Meltem Kurt Yüksel, Özlem Doğan.

**Methodology:** Merve Yüksel, Meltem Kurt Yüksel.

**Validation:** Merve Yüksel, Özlem Doğan, Meltem Kurt Yüksel, Merve Aydoğan.

**Writing – original draft:** Merve Yüksel, Meltem Kurt Yüksel.

**Writing – review & editing:** Merve Yüksel, Meltem Kurt Yüksel, Merve Aydoğan.
